# Label-Free MicroRNA Optical Biosensors

**DOI:** 10.3390/nano9111573

**Published:** 2019-11-06

**Authors:** Meimei Lai, Gymama Slaughter

**Affiliations:** Frank Reidy Research Center for Bioelectrics, Department of Electrical and Computer Engineering, Old Dominion University, Norfolk, VA 23508, USA

**Keywords:** microRNA, nanomaterials, label-free, optical sensor

## Abstract

MicroRNAs (miRNAs) play crucial roles in regulating gene expression. Many studies show that miRNAs have been linked to almost all kinds of disease. In addition, miRNAs are well preserved in a variety of specimens, thereby making them ideal biomarkers for biosensing applications when compared to traditional protein biomarkers. Conventional biosensors for miRNA require fluorescent labeling, which is complicated, time-consuming, laborious, costly, and exhibits low sensitivity. The detection of miRNA remains a big challenge due to their intrinsic properties such as small sizes, low abundance, and high sequence similarity. A label-free biosensor can simplify the assay and enable the direct detection of miRNA. The optical approach for a label-free miRNA sensor is very promising and many assays have demonstrated ultra-sensitivity (aM) with a fast response time. Here, we review the most relevant label-free microRNA optical biosensors and the nanomaterials used to enhance the performance of the optical biosensors.

## 1. Introduction

MicroRNAs (miRNAs) are endogenous tiny regulatory (~19–24 nucleotides in length) RNA species found across plants, animals and humans. They play a critical role in the regulation of gene expression by binding to mRNA targets to promote their degradation or repress their translation [1,2]. After the discovery of the first miRNA in *Caenorhabditis elegans* in 1993 [3], a new era in molecular biology arose and numerous studies have since reported on their mode of biogenesis and biological functions. A little over 2600 unique miRNAs have been discovered in humans [4], animals [5], plants [6], and viruses [7,8]. Nearly all diseases, including diabetes [9,10,11], cardiovascular [12], cancer [13,14,15], fibrosis [16,17,18,19], immunological [20] and neurodegenerative disorders [21,22,23] have been linked to an aberrant number of miRNAs, misregulated miRNA signal pathways [24], or distinctive miRNA profiles [25]. It has been shown that these miRNAs play an important role in numerous cellular processes and diseases, and are well preserved in a variety human specimens, such as tissue, blood, or urine [26,27,28,29]. Further, miRNAs have been shown to be measurable with a high degree of sensitivity and are therefore ideal biomarker candidates in disease diagnosis when compared to traditional protein biomarkers that can be easily degraded over time [30,31,32]. However, the accurate detection and quantification of miRNAs remains a big challenge in the field of biosensing due to the current limitations of the analytical tools available [32,33,34,35,36,37]. Conventional techniques used for assaying miRNAs [32,38], such as northern blotting [39,40], quantitative reverse transcriptase polymerase chain reaction (qRT-PCR), and cDNA microarrays [41,42,43] are extremely complicated, time-consuming, laborious, cost-ineffective and exhibit poor sensitivity. These challenges are attributed to the intrinsic properties of miRNAs such as their low mass, short sequence length, high sequence similarity, low abundance (∼0.01% of the total mass), and only a few molecules per cell [44,45,46]. 

To date, optical based approaches are the most popular techniques in literature used for detecting miRNAs and are widely studied in the development of biosensors [47,48,49,50]. Optical fluorescence-based biosensors [46,51,52] that detect the hybridization between the miRNAs and their respective complementary mRNA probes have been shown to be highly sensitivity using fluorescence spectroscopy [53]. Although the use of fluorescent labeled miRNAs in the hybridization with the immobilized probes generates a fluorescent signal that correlates with the presence of the target miRNAs, this technique can result errors for the non-target detections and thereby impacting the specificity of the biosensor. Precise labeling of each biomolecule results in a time-consuming process and, usually, the labeling may further affect the function of the biomolecule. Additionally, it is very difficult to quantify the captured miRNAs since the number of fluorophores per miRNA molecules cannot be precisely controlled thereby resulting in a signal bias in the fluorescence intensity.

The label-free detection of biomolecules has been a long-standing goal in the development of optical biosensors [48,49,54]. Label-free miRNA biosensors employ target miRNA biomolecules in their natural state and are unlabeled or unmodified. The detection mechanism depends on the measurement of the change in the intrinsic physical parameter of the biosensor, therefore resulting in a cost-effective, more reliable, easy and faster detection of the biorecognition interaction in a real-time. The physical parameter used in most label-free refractometric sensing devices is the index of refraction [55]. The binding event induces a change in the index of refraction near the biosensor surface and this biorecognition interaction is corelated to the biomolecule concentration. Label-free optical biosensors have attracted a significant amount of intensive investigations in recent decades due to their ability to employ ultra-small detection volume while achieving high sensitivity and low limit of detection (LOD) in real-time. These characteristics enable label-free optical biosensors to be advantageous over fluorescence-based biosensors since label-free biosensor signal does not depend on the overall quantity of biomolecules in the sample detection volume. 

Nevertheless, optical based methods are essential to achieve a robust, multiplexed analysis of miRNA with high sensitivity and specificity and a large linear dynamic range. Optical biosensors are highly desirable for detecting the interactions between biomolecules and have become more versatile than other types of sensing technologies. In the following, we review some of the most relevant miRNA label-free optical biosensor detection platforms, namely surface plasmon resonance (SPR) based biosensors, interferometer-based biosensors, and whispering gallery mode (WGM) microresonator-based biosensors, and the strategies used to detect ultralow concentrations of the target miRNAs with and without an amplification strategy.

## 2. Surface Plasmon Resonance (SPR) Biosensors

Surface plasmon resonance (SPR) was initially applied to biosensing by Liedberg et al. [55]. Since SPR biosensors have been widely used to detect various chemical and biological species such as cells, bacteria, peptides, nucleic acids, proteins and viruses, they have become a very important instrument for studying the interactions between target and biorecognition molecules [56,57,58,59,60]. SPR have also gained significant attention in both fundamental research and industry after its commercialization in 1990 by Biacore (GE Health care). Currently, SPR biosensors are commercialized by many companies all over the world [61] and have recently gained attention for miRNA detections [25,30,45,62,63,64]. The principle and applications of SPR biosensors have been well reviewed [56,57,60]. Here, we will limit the scope to label-free detection using SPR biosensors.

**Figure 1:**
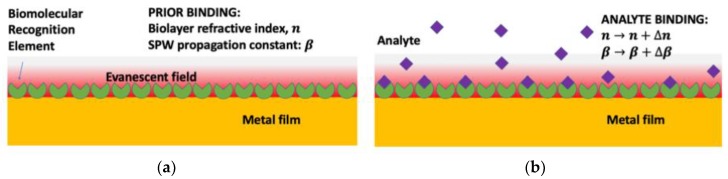
Principle of surface plasmon resonance (SPR) sensor: (**a**) prior to binding and (**b**) analyte binding where the number of analytes captured results in a change in the index of refraction (RI) near the surface of the metal layer.

Using SPR technology as an optical platform for biosensing, a surface plasma wave (SPW) that is propagated along the metal-dielectric interface layer decays exponentially into the interfacing bulk solution as the evanescent field (Figure 1a) [56,60,65,66]. The SPW propagation constant, β, is very sensitive to the changes in the refractive index (RI) of the dielectric since a large portion of the SPW is concentrated in the dielectric layer. The biorecognition event results in an increase in the local RI, n, near the surface of the metal film (Figure 1b) that is optically detected as the transduction signal.

**Figure 2:**
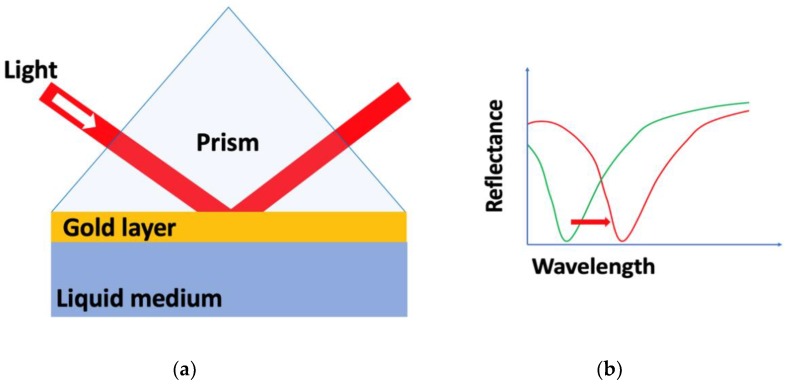
(**a**) Surface plasmon resonance biosensor based on the attenuated total reflection (ATR) method and (**b**) spectrum shifts observed before and after change in the refractive index as the result of a biorecognition event.

The three most common methods used to excite the SPW [60] are prism coupling [67], waveguide coupling and grating coupling, wherein prism coupling with the attenuated total reflection (ATR) method (Figure 2a) has received significant attention [68,69,70,71,72,73]. A change of the propagation constant as a result of the biorecognition event induces a change in the coupling wavelength and hence a dip shift is observed (Figure 2b) using the ATR method.

**Figure 3:**
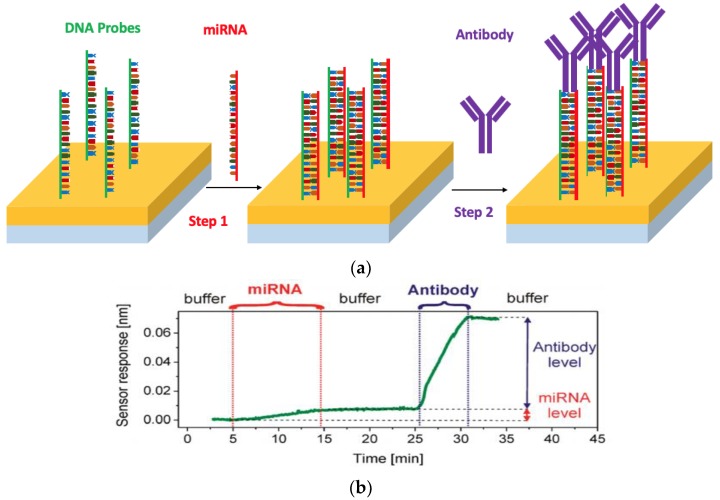
(**a**) Schematic representation of the antibody-based assay for detection of miRNA-122. (**b**) Sensor response to the miRNA and antibody. Reproduced with permission from [71]. Copyright American Chemical Society, 2010.

**Figure 4:**
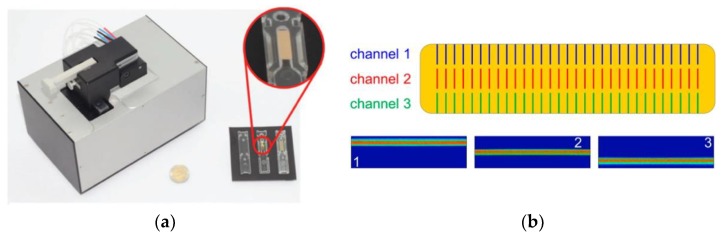
(**a**) Fraunhofer compact SPR biosensor (left) with chips and flow cells (right) and (**b**) schematic representation of the arrangement of the three 1D spot arrays (channel 1, channel 2 and channel 3) on the gold surface illuminated by three side-by side distinct stripes lights (1, 2 and 3). Reproduced with permission from [25,72]. Copyright John Wiley and Sons, 2011 and Walter De Gruyter GmbH, 2016.

Several SPR-based approaches are based on microfluidic techniques for the detection of miRNA. A novel label-free portable SPR biosensor has been developed by Sipova et al. [71] for fast and sensitive detection of miR-122 acquired from mouse liver tissue. A DNA–RNA hybrid antibody-based assay was employed to bind to the DNA–RNA hybrid structure. Figure 3a provides a schematic illustration of the surface modification of the electrode, wherein the first step involved the modification of the sensor surface with DNA probes to capture the target miRNA. In the presence of the analyte, the miRNA is captured and is subsequently recognized by the antibody that has an affinity to the DNA–RNA hybrids by binding to the miRNA–DNA duplex as shown in Step 2 of Figure 4a. The captured miRNA signal was significantly enhanced by the binding of the antibody to the miRNA–DNA duplex as depicted in the corresponding response (Figure 3b). In addition, the sensitivity of the assay was dramatically increased as a result [74,75]. The LOD for the direct detection of miR-122 was 100 pM and 2 pM LOD was observed using the antibody-amplified strategy. The SPR biosensor was further validated for miR-122 extracted from mouse liver tissue and the results agreed with those reported by qPCR.

Schmieder et al. [25] presented an optical system comprising a disposable SPR chip and microfluidic hybrid system as shown in Figure 4a. The system is coupled with a lateral imaging system to enable parallel analysis of three 1D spot arrays illuminated by three light channels on the gold surface (Figure 4b) to detect the target biomolecule. The disposable polymer-based biosensor chips (60 mm × 13 mm) were fabricated using injection molding technology and packaged to allow easy handling. The biosensor surface was sputter coated with a thin gold layer (45 nm) via magnetron sputtering [72]. The chip surface was functionalized with thiol-modified oligonucleotide probes to enable the hybridization of a locked nuclei acid oligo (LNA-93) to miRNA-93. LNA are typically used to enhance the probe affinity for its target among other functions. This approach provides high sensitivity and specificity. The target miRNA-93 was successfully detected using this label-free strategy with a limit of detection (LOD) of 10 nM. The LOD was further enhanced using an amplification step similar to Sipova et al. [71], wherein an RNA–DNA-hybrid antibody, which only binds to RNA–DNA double strands on the sensor surface, was employed to bind to the RNA–DNA hybrid structure. Therefore, upon successful hybridization of miRNA-93 with LNA-93 probe, the antibody attached to the hybridized structure and significantly enhanced the signal of the sensor with an improved LOD of 10 pM within 30 minutes. In this approach, the LNA probes were demonstrated to be successful in enabling the direct label-free detection of miRNA-93 and signal amplification was achieved using RNA–DNA-hybrid antibody. This signal amplification strategy yields excellent results, and many studies have employed similar amplification method (Table 1) whereby the overall mass or size of the target biomolecule is increased via bioconjugation to other biomolecules or nanoparticles, and hence a significant enhancement of the signal [45,75,76,77,78].

**Figure 5:**
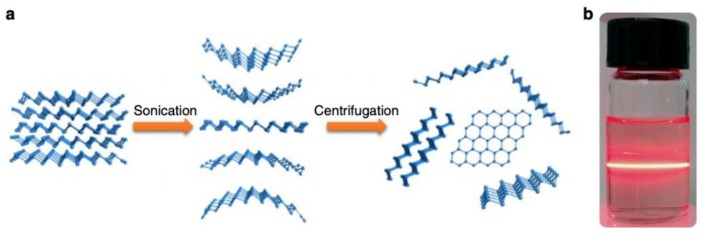
(**a**) Preparation of two-dimensional antimonene nanosheets. (**b**) Faraday–Tyndall effect used to verify the existence of antimonene nanosheets in the solution. Reproduced with permission from [44]. Copyright Springer Nature, 2019.

More recently, a SPR biosensor based antimonene was fabricated by Xue et al. [44] for the label-free detection of miRNA-21 and miRNA-155. Antimonene is a two-dimensional nanomaterial with delocalized 5*s*/5*p* orbitals and honeycomb lattice that results in a much stronger interaction with ssDNA when compared to graphene-based DNA SPR biosensors. The synthesis of two-dimensional antimonene nanosheet materials is shown in Figure 5a. A few layers of antimonene nanosheets were obtained via sonication liquid-phase exfoliation [80], wherein the weak van der Waals forces were broken and the presence of antimonene nanosheets in the solution were confirmed by the Faraday–Tyndall effect [81] as shown in Figure 5b. The lateral dimensions of the antimonene nanosheets used were greater than 300 nm with a thickness of approximately 3 nm.

**Figure 6:**
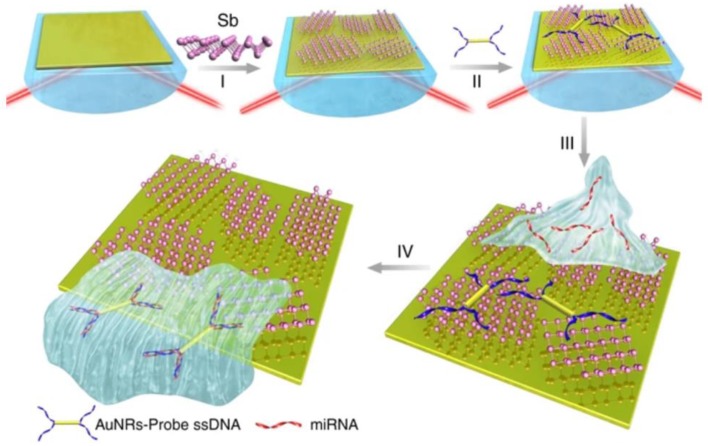
Fabrication of the microRNA sensor integrated with antimonene nanomaterials. (I) The antimonene nanosheets casted on Au substrate. (II) AuNR-ssDNAs adsorption. (III) miRNA binds to complementary AuNR-ssDNA to form a double-strand with complementary AuNR-ssDNA. (IV) AuNR-ssDNA released from the antimonene nanosheets. Reproduced with permission from [44]. Copyright Springer Nature, 2019.

Figure 6 provides a schematic illustration of the antimonene-modified SPR biochips. The antimonene nanosheets were assembled on gold film using a layer-by-layer (L = 4) approach followed by the adsorption of gold nanorods (AuNR) modified ssDNAs. The formation of the AuNR-ssDNA complex was necessary to amplify the SPR signal through the extra mass added via the AuNRs. Various concentrations of miRNA were introduced to hybridize with complementary AuNR-ssDNA adsorbed on the antimonene surface. The interaction between miRNA with AuNR-ssDNA resulted in the formation of a double stranded DNA, which has a low affinity to antimonene, thereby causing the hybridized target miRNAs to be released from the antimonene surface and resulting in a shift in the SPR signal. The AuNR–ssDNA-modified antimonene SPR biosensor exhibited a LOD of 10 aM. Compared to existing microRNA sensors, this was a 2.3–10,000 times decrease in the limit of detection of miRNA biosensors (Table 1) and the highest sensitivity described thus far in an optical based miRNA detection system.

This antimonene based biosensor is the first SPR biosensor to use of two-dimensional antimonene materials for clinically relevant miRNA detection. The application of antimonene is unique as it can release the target biomolecules from the surface rather than retaining the miRNA on the biosensor surface, as is the case for most SPR sensors. However, the impact of complex biological matrices on the miRNA detection performance was not demonstrated with this SPR biosensor.

Overall, these results demonstrate that SPR biosensors can detect miRNAs. Despite the improved sensing performance observed, the incorporation of an amplification step complicates the overall biorecognition process, in addition to an increased in the analysis time. A total analysis time for miRNA detection of approximately 30 minutes was achieved by Sipova et al. despite the additional antibody (amplification) step and aM LOD was achieved using the two-dimensional antimonene nanosheets by Xue et al. To improve the performance of the SPR sensors, other techniques, such as SPR imaging (SPRi) [62], localized surface plasmon resonance (LSPR) [44,63,64], magneto-optical surface plasmon resonance (MO-SPR) [82], long-range surface plasmon (LRSP) [83], and surface enhanced Raman scattering (SERS) [84,85], have been integrated with SPR to achieve high sensitivities, as well as to provide an extension to the highly multiplexed sensing architecture.

### 2.1. SPR Imaging (SPRi)

**Figure 7:**
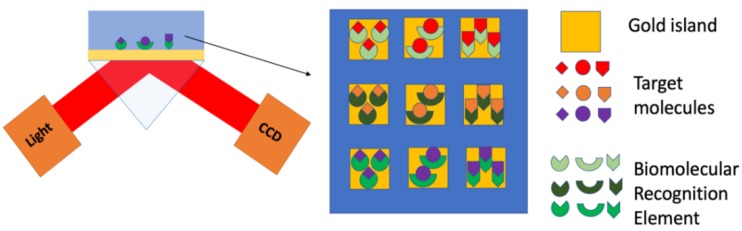
Illustration of the reflected light from the biorecognition event on the array being detected via a CCD camera for each array spot as the change of intensity of the reflected light.

Recently, SPRi and LSPR have gained significant attention in the area of miRNA biosensing. SPRi is a label-free, high-throughput SPR biosensor (Figure 7) designed for measuring biomolecular events simultaneously using an array of biomolecular elements chemically patterned on metallic surfaces [57,86,87,88]. Compared with SPR, SPRi uses a CCD camera to acquire the SPR images and the sensorgrams of the arrayed surface. Since the angle and wavelength of the propagating light are fixed, the differences in the light intensities reflected in each array spots are correlated with the distribution of the RI at the surface of the metal film. By this means, different biorecognition events can be detected simultaneously in real time.

**Figure 8:**
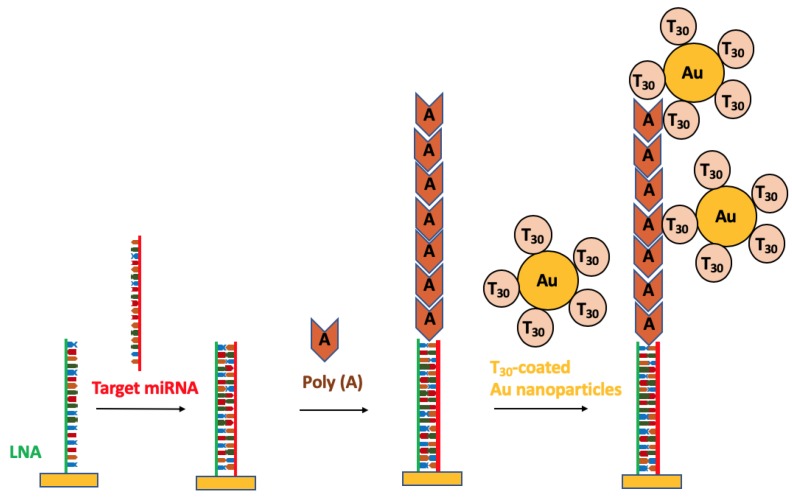
miRNAs detection mechanism using a combination of surface polyadenylation chemistry and nanoparticle amplified SPRi detection.

A variety of strategies employing nanoparticles have been developed for the detection of miRNAs in order to reduce the artifacts generated from the labeling and amplification steps. Recently, Fang et al. [77] employed a nanotechnology-based strategy using polyadenylation (poly(A) polymerase enzyme) chemistry combined with gold nanoparticle-amplification for SPRi detection of miRNAs from mouse liver tissue. Figure 8 illustrates the detection mechanism employed for the identification of miRNAs. LNAs were immobilized on the surface of the sensor to form LNA microarrays. The target miRNAs were then introduced to hybridize with the LNA capture probes on the sensor surface. Poly(A) tails were subsequently added to the surface bound miRNAs via the poly(A) polymerase surface reaction. After this, the T_30_-coated AuNPs were added and allowed to hybridize with the poly(A) tails to amplify the signal and enable the subsequent detection of the miRNAs via SPRi. This methodology provided an ultrasensitive detection of miRNA through the combination of a surface-based enzyme amplification and DNA-coated nanoparticle strategy [86,89,90,91]. A high sensitivity was achieved at fM levels as the result of the strong hybridization afforded between the LNA-miRNAs and the poly(A) tails-DNA-coated AuNPs. The 10-fold increase in the sensitivity of the microarray assays achieved using this procedure is an improvement over the fluorescence based microarrays strategies currently employed [92]. The nanotechnology-based assay was successfully demonstrated to quantify three miRNA sequences from mouse liver: miR-16, miR-23b and miR-122b in a range from 20 fM to 2 pM with a detection limit of 10 fM in total RNA sample [77].

**Figure 9:**
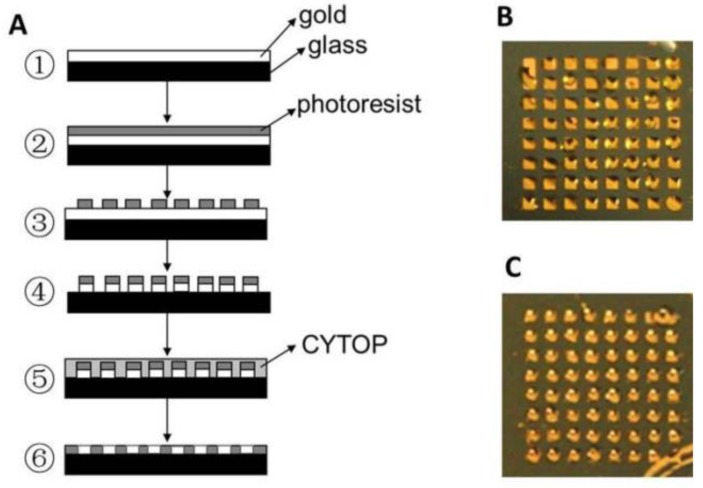
(**A**) Fabrication procedure for fabricating gold-islanded biochip with hydrophilic/hydrophobic spacer: ① gilding, ② photoresist spin-coating, ③ patterned exposure and developing, ④ gold etching, ⑤ CYTOP spin-coating and baking, ⑥ stripping excessive CYTOP and photoresist; (**B**) Image of the chip wetted by water; (**C**) Image of the chip dropped with hydrophilic samples. Reproduced with permission from [75]. Copyright American Chemical Society, 2017.

Hu et al. [75] fabricated an innovative SPRi sensing biochip that incorporates in-plane and vertical signal amplification strategy for the direct detection of a multiple tumor biomarker, miRNA-15a at sub-fM levels. The main idea behind the orthogonal signal amplification is to allow for additional mass to be added to the target spot in-plane and upward from the sensor surface, thereby fully optimizing the use of the 3D space for sample interrogation. The sensing biochip was fabricated with isolated gold islands bordered by hydrophobic CYTOP (Figure 9) in order to construct 3D space for biomolecular interactions. A 1.0 cm × 1.0 cm glass slide was coated with thin layer of chromium/gold (2 nm/48 nm), which was processed using standard photolithography techniques to realize gold islands surrounded by fluoropolymer CYTOP passivation layer. The direct detection of miRNA was realized by coupling the miRNA-initiated cyclic DNA–DNA hybridization reaction along surficial direction on the target probe with the DNA-initiated upward cyclic polymerization reaction. The unique aspect of this work was that the amplification operation occurred in-plane and in the vertical directions. Since each direction can have a 10^3^-fold increase in the enhancement of the signal, a total of two directions provides a net increase of 10^6^-fold for the signal enhancement. This amplification method utilizing the two dimensions resulted in more mass being bound to the surface of the target sample. This approach provides an effective use of the 3D space on and above a sample spot, thereby achieving a LOD and limit of quantification down to 0.56 fM and 5 fM for miRNA-15a, respectively. This technique resulted in about 10^7^-fold increase in the sensitivity compared to traditional SPRi miRNA detection. This strategy was further validated by quantifying miRNA-15a in blood samples from a healthy and cancer patient. The results were found to be similar to those reported for colorectal cancer tissues.

Compared with the traditional SPR sensors, SPRi chips incorporating metallic nanostructures and microfluidics make these optofluidic devices ideal platforms for profiling miRNA in biological samples. The signal amplification strategies employed have been proven to increase the detection signal and the sensitivity. Furthermore, the used of an orthogonal signal amplification, which employs two signal amplification steps was shown to dramatically increase the detection signal by adding more mass onto the target miRNA. The use of a secondary signal amplification strategy resulted in an improvement in the LOD and therefore demonstrated the potential to be implemented in diagnostic and point-of-care (POC) applications. Therefore, SPRi biosensors have emerged as one of the most promising optical sensing techniques to meet the demands for the implementation of lab-on-a-chip technologies [89].

### 2.2. Localized Surface Plasmon Resonance (LSPR)

**Figure 10:**
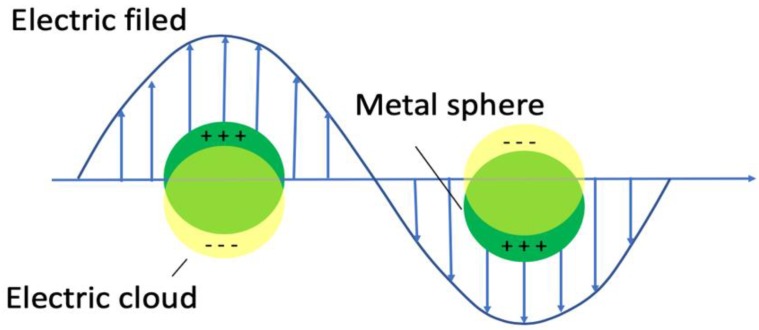
Schematic diagram for a localized surface plasmon excited in a small metallic particle.

With the recent development of nanotechnology and the manipulation of metallic structures on the nanoscale, LSPR spectroscopy continues to garner attention and has demonstrated several advantages beyond the traditional SPR due to their geometric dependent LSPR properties [90,91,92]. In brief, localized surface plasmons occurs when a light wave interacts with metallic nanoparticles (NPs) that are much smaller than the incident wavelength (Figure 10) [93]. The interaction gives rise to the free electrons in the metallic material to interact with the incident light and oscillate in phase with the incident electric field, thus generating a localized surface plasmon resonance. At resonance, the total electric field amplitude near the NPs can be increased substantially compared to the incident amplitude. In addition, the resonant frequency is highly correlated to the properties of the NPs, such as the NPs spatial distribution, chemical composition, size, geometry, and dielectric environment. These properties have been explored in the development of biosensors where the biorecognition events between the target analyte and the nanostructures modified with biorecognition elements result in the modulation of the refractive index and a peak shift in the LSPR spectra.

**Figure 11:**
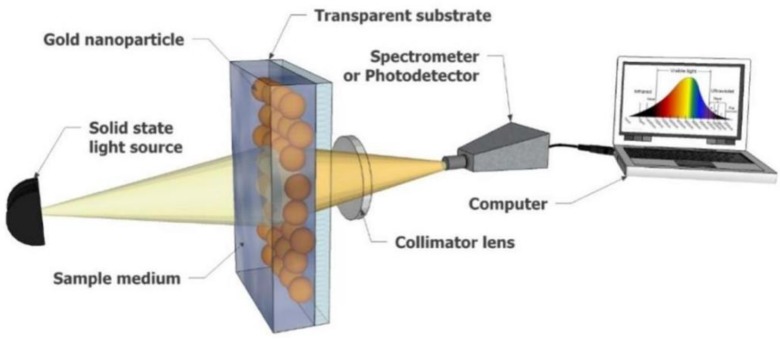
LSPR-based sensor using transmission attenuation configuration. Reproduced with permission from [94]. Copyright MDPI, 2018.

Although there are many different configurations for generating LSPR spectra (transmission, reflective geometry, dark-field scattering, etc.) [64], the most straightforward configuration is the transmission-based spectroscopy shown in Figure 11. This configuration enables the LSPR biosensor to monitor the extinction spectrum of the light passing through the sample without the use of prism, thus making it a cost-effective approach that enables miniaturization [93,94]. In a study motivated by the development of a direct hybridization strategy for detecting miRNAs in circulation pancreatic cancer patients that is cost-effective, Joshi et al. [62] developed and demonstrated the first solid-state ultrasensitive LSPR biosensor for microRNA that is both label-free and amplification-free. 

The biosensor explored the unique properties of anisotropically shaped metallic nanostructure (gold nanoprisms) by monitoring the LSPR dipole peak (λ_LSPR_) [62,63] in physiological media (human plasma). The gold nanoprisms were on average ∼35 nm in size (edge length) and exhibited an unprecedentedly reversible 21 nm shifting towards longer wavelengths of the LSPR peak as a result of the 0.6 nm thickness increase in the local dielectric environment [62]. The electromagnetic fields were dramatically enhanced at the nanoprism’s sharp tips, which improved the enhancement of the Raman scattering intensity of the analytes. 

**Figure 12:**
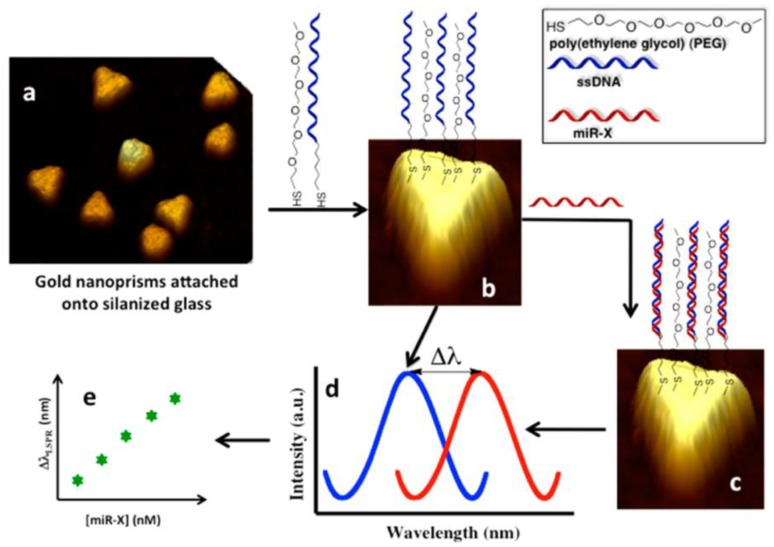
Plasmonic biosensors based on nanoprisms: (**a**) Chemically synthesized gold nanoprisms covalently attached onto glass coverslip via 3-mercaptopropyltriethoxysilane-functionalized. (**b**) Surface modification of gold nanoprisms equimolar mixture of SH-C6-ssDNA-X and PEG6-SH in PBS buffer (pH 7.4). (**c**) Incubation of sensor in miR-X solution and formation of DNA duplex. (**d**) Extinction spectrum of the surface modification with SH-C6-ssDNA-X and PEG6-SH (blue curve) and after incubation with miR-X (red curve). (**e**) Plot of Δλ_LSPR_ versus log of miR-X concentrations. Reproduced with permission from [62]. Copyright American Chemical Society, 2014.

Figure 12 illustrates the mechanism of the direct hybridization strategy enabled by the plasmonic biosensor fabricated by Joshi et al. [62]. The gold nanoprisms depicted in Figure 12a were chemically synthesized and displayed an optimal λ_LSPR_ at ∼797 nm, which is well suitable for physiological media such as blood and plasma. The gold nanoprisms were attached to a silanized glass support platform followed by chemical functionalization with poly(ethyleneglycol)-6-thiols (PEG6-SH) and the complementary probes (-C6-ssDNA). The PEG6-SH and ssDNA formed a self-assembled monolayer (SAM) of biorecognition elements that provided the required space for hybridization with complementary miRNAs (Figure 12b). The PEG thiols were used as spacers to increase the selectivity of the sensor by minimizing nonspecific adsorption and are not reactive toward miRNAs or other biological components found in plasma. Upon the direct hybridization of the target miRNAs (miR-21 and miR-10b) to their complementary ssDNA, a shift in the λ_LSPR_ to longer wavelength was observed in the biosensor response (Figure 12c). The total shift (Δλ_LSPR_) observed was dependent on the miRNA concentration (Figure 12d) and is used to determine the LOD (Figure 12e). The calculated LODs for miR-21 in three different biological samples were found to be between 23−35 fM. This is more than a 1000-fold decreased in the LOD when compared to label-free microring resonators [73] (Table 1) and the nanopore based miRNA sensors [95], which employ an advanced fabrication procedures. 

**Figure 13:**
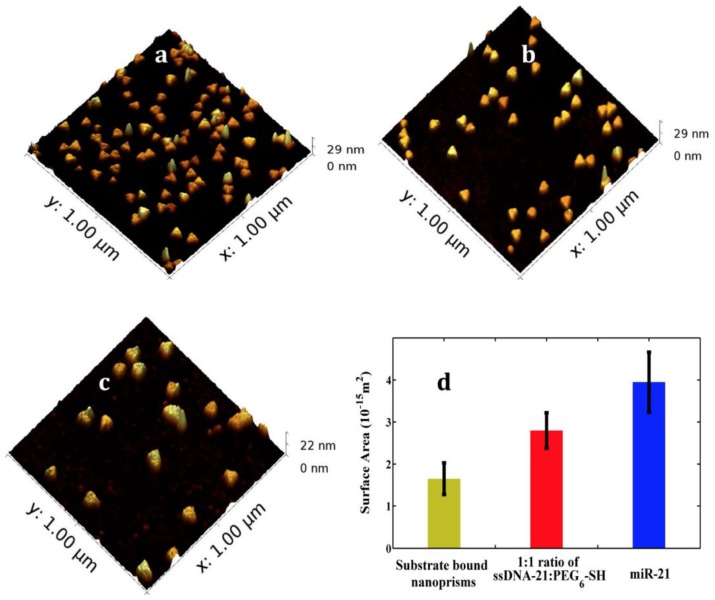
Surface modification of LSPR sensor. (**a**) AFM micrographs after functionalization with 1:1 ratio of HS-C6-ssDNA-21/PEG6-SH (**b**) and after hybridization with 100 nM miR-21 in 40% human plasma. (**c**) bare gold nanoprisms. (**d**) plot of the change in surface area of the gold nanoprisms after each surface modification. Reproduced with permission from [62]. Copyright American Chemical Society, 2014.

Figure 13 provides the AFM micrograph of the change in surface area as a result of miR-21 hybridization with mixed ssDNA-21 and PEG6-SH-functioanlized gold nanoprisms. The attachment of miRNAs to the sensor surface increased the thickness of the dielectric environment locally and therefore impacting the RI of the LSPR. The modulation in the LSPR properties resulted in the realization of an ultrasensitive label-free biosensor. A DNA-RNA cleaving enzyme, RNase H was used to break the bonds between the DNA-RNA duplex in order to regenerate the biosensor surface to allow for multiple assay cycles. This LSPR biosensor detected miRNA-21 and miRNA-10b in pancreatic cancer patient plasma at sub-fM concentration and miRNA-10b at the aM concentration level in pancreatic cancer cell lines, culture media and plasma. This plasmonic based biosensor could detect miRNAs in human plasma without sample preparation and thereby enabled the first label-free and amplification-free detection of miRNAs in complex, clinically relevant samples.

## 3. Integrated Planar Optical Waveguide Interferometer Biosensors

**Figure 14:**
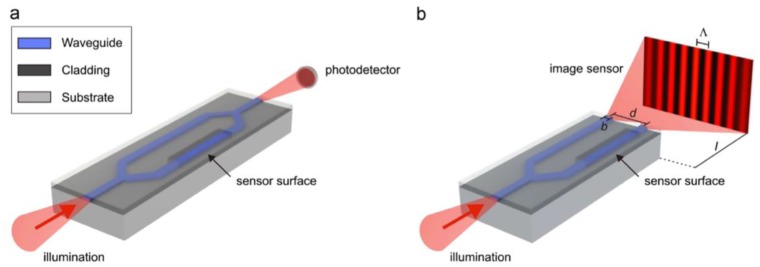
(**a**) Mach–Zehnder interferometer and (**b**) Young interferometer biosensor configurations. Reproduced with permission from [96]. Copyright Elsevier, 2014.

Integrated planar optical waveguide interferometer biosensors combine waveguiding and interferometry approaches to measure the optical phase difference between the reference arm and the probing arm by monitoring the interaction of the target molecules with evanescent field near the surface of the sensor [79,96]. The Mach–Zehnder and Young interferometers illustrated in Figure 14a,b, respectively, are extremely sensitive, exhibit extended dynamic range and interaction length when compared with traditional SPR for changes in refractive index and surface mass density. However, their integration is technologically complex [97] in terms of multiplexing.

**Figure 15:**
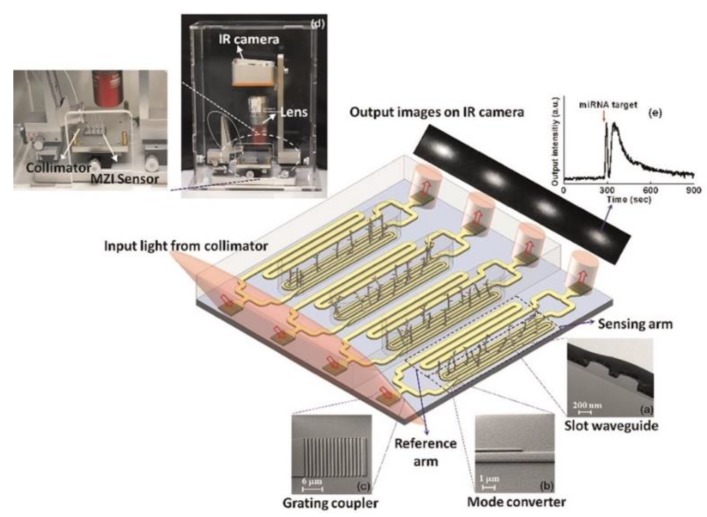
Schematic diagram of the MZI biosensor platform (**a**) TEM image of the cross section of the waveguide. (**b**) SEM images of mode converter, and (**c**) a silicon nitride grating coupler. (**d**) Photograph image of MZI biosensor. (**e**) Output intensity displaying a 4πphase shift after the target miRNA recognition. Reproduced with permission from [31]. Copyright Elsevier, 2015.

A Mach–Zehnder interferometer (MZI) based miRNA detection system was fabricated by Liu et al. [31] for the label-free detection of multiple miRNAs in clinical relevant samples. The MZI biosensor was fabricated using standard complementary metal-oxide-semiconductor (CMOS) fabrication process [97,98], where the waveguides and gratings were patterned on silicon wafer with Si_3_N_4_ and oxide layers using deep UV lithography and reactive ion etching, followed by plasma enhanced chemical vapor deposition of silicon oxide cladding layer. The biochip contains four identical MZI sensors (Figure 15) with the waveguide splitting into two arms (reference and sensing arms) which recombine at the opposite end to enable the multiplexed detection of miRNAs. The synthetic target probes for target miRNAs was covalently immobilized on the amine functionalized biosensor surface and the target miRNA in PBS solution was added via microfluidics to hybridize with the DNA probe captured on the MZI biosensor surface. The hybridization reaction resulted in a phase change that was measurable in terms of change in light intensity. Oligoribonucleotides and oligonucleotides were used as the target probes for synthetic miR-21, let-7a and single nucleotide polymorphism of the let-7 family of miRNAs. A LOD of 1 nM was achieved with the MZI system. The MZI miRNA-based biosensor was successfully demonstrated in human urine samples to validate its clinically capability as a point-of-care device; however, the LOD was not as low as previously reported for other biosensing system (Table 1). The disposable MZI biochip is designed to be cost-effective, label-free, and eliminates the need for temperature control or sophisticated detection system. Although the LOD was not as impressive as other optical based biosensors, it detected the miRNA within 15 minutes and the LOD can be improved by increasing the interaction length for biorecognition events, or by reducing noise levels in the system.

**Figure 16:**
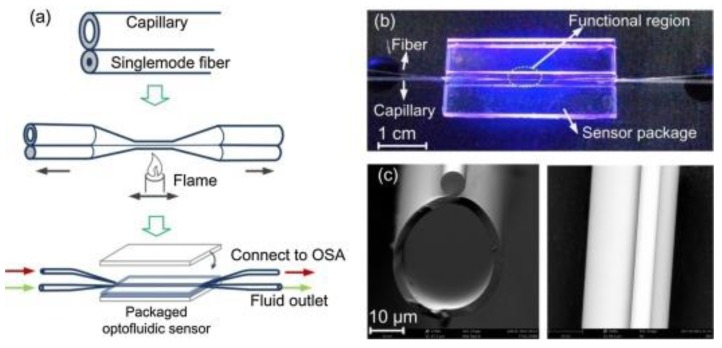
Microfiber-capillary optofluidic sensor fabricated by Liang et. al (**a**) Schematic diagram of the fabrication process of the sensor. (**b**) Image of the sensor platform. (**c**) SEM image of the cross-section and side view of the sensor. Reproduced with permission from [79]. Copyright Elsevier, 2017.

A unique interferometer biosensor based on microfiber capillary optofludic biosensor was recently reported by Liang et al. [79] to explore the interference spectrum during the biorecognition event between DNA and miRNA. The combination of the microfiber and a capillary aligned and tapered together formed what is known as a modal interferometer depicted in Figure 16. Figure 16a depicts the fabrication process for the biosensor and Figure 16b,c provides the optical image and scanning electron micrograph (SEM) of the as-fabricated biosensor, respectively. The biorecognition events occur at the interior surface of the capillary and in turn induces the interference spectrum shift with the pre-immobilization of DNA probes. The optofluidic biosensor was demonstrated to detect miRNA-let7a with a log-linear range of 2 nM to 20 μM and a LOD of 212 pM without an amplification step. The biosensor was also demonstrated to be capable of discriminating mismatches in miRNAs, which is a desired characteristic for miRNA profiling. Additional benefits offered by the optofluidic miRNA biosensors is that it is amplification-free, portable, simple to fabricate and handle, thereby enabling future applications beyond the benchtop.

## 4. Whispering Gallery Modes (WGMs) Resonators Biosensors

**Figure 17:**
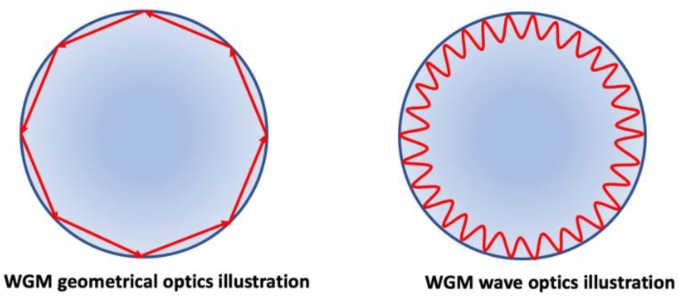
WGMs supported upon total internal reflection can be understood by either geometrical optics (**left**) or wave optics (**right**).

Optical biosensors based on whispering gallery mode (WGM) microresonators have attracted significant attention in the last two decades due to their high sensitivity and low LOD [92,99,100]. An optical WGM resonator is a microstructure with smooth edges, such as microspheres, microrings, microtoroids, microdisks, microbottles, microcapillaries and microbubbles, etc., and are made of dielectric materials, with a relatively larger refractive index compared to the surrounding environment. Due to the total internal reflection of the cavity surface, light can be confined in the resonator structure as depicted in Figure 17. In general, the radius of the WGM resonator, R is much larger than the resonant wavelength λ, i.e. R >>  λ, to the first order approximation, and the resonance condition happens when:

$$m\lambda =2\pi rn_{eff}$$

where *m* is an integer, *r* is the outer radius of the resonator, and *n_eff_* is the effective index. In another word, when the total length of a trip around the cavity equals an integer number of wavelengths, the system is exactly on resonance. When a target molecule is captured on the surface of the resonator, the WGM spectral position shifts. The kinetic information of the binding events near the surface of the WGM resonator can be obtained by monitoring the WGM spectrum directly or indirectly.

There are three main sensing mechanisms for WGM resonator: mode shifting [101], mode splitting [102], and mode broadening [103]. Mode shift refers to the changes that occur in the resonant wavelength of the resonator in the presence of the analyte of interest and is the most commonly used sensing mechanism for WGM resonators, due to its broad applicability to a range of analytes. A key major disadvantage associated with mode shift is that it is susceptible to changes in ambient conditions such as temperature, pressure, and humidity, and this tends to introduce unwanted drift in the mode shift signal. However, mode shift sensing has been used to detect changes in physical parameters surrounding WGM resonator, such as heat [104,105], pressure [106], and magnetic fields [107]. Both mode splitting and mode broadening are self-referenced detection mechanisms which can remove the influence of the environment temperature or system instability disturbance and typically involve the adsorption of nanoparticles or a single biomolecule on the resonator surface.

**Figure 18:**
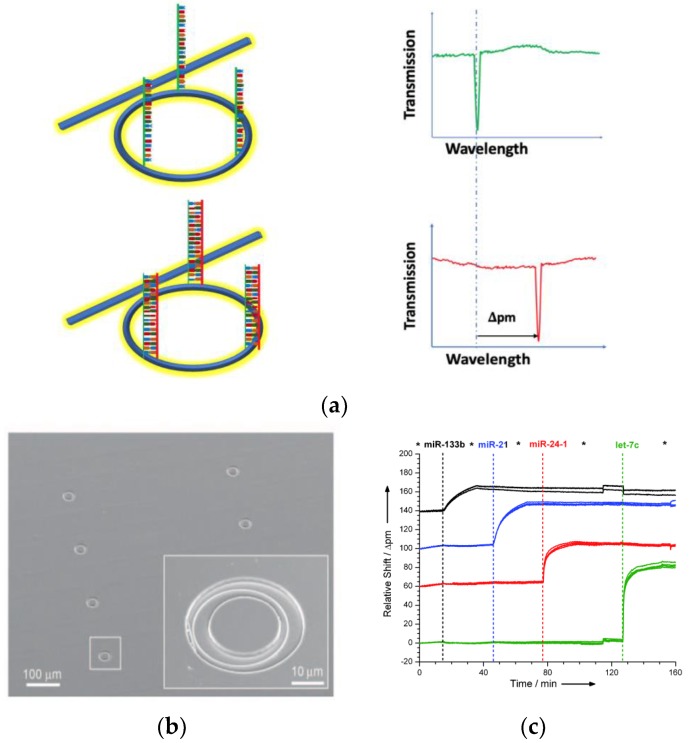
Schematic diagram of the microring biosensor system for miRNA detection. (**a**) The microring is functionalized with a capture DNA sequence (green curve). The capture of the target miRNA (purple curve) causes a shift in the wavelength required to achieve optical resonance. (**b**) SEM image showing six microrings on a sensor array chip. (**c**) The wavelength shift after miRNA recognition of the selected miRNAs. Reproduced with permission from [73]. Copyright John Wiley and Son, 2010.

**Figure 19:**
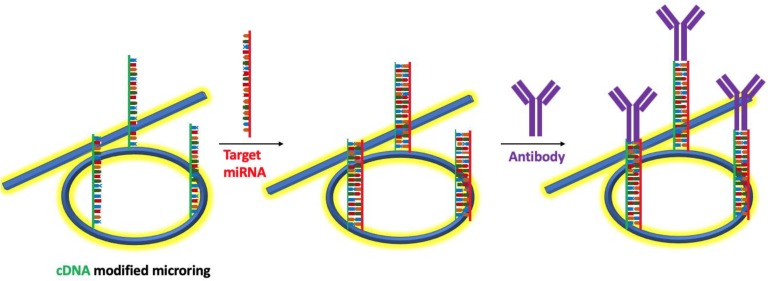
Schematic diagram of the Anti-DNA-RNA amplification experiment. A complementary DNA modified microring is exposed to target miRNA followed by the anti-DNA-RNA antibody for signal amplification.

Qavi et al. [73] reported a label-free detection system using commercially available multiplexable array of microring resonators fabricated using silicon-on-insulator to analyze four miRNAs simultaneously as shown in Figure 18a. The microring resonator system contained a total of 32 independently addressable microrings (ϕ = 30 μm) coated with a fluoropolymer cladding layer. Figure 18b shows a SEM micrograph of the sensor array. The oxide-coated microring resonator were covalently modified with ss-DNAs probes after which the target miRNA was flowed over the sensor surface to hybridize to the complementary ss-DNA. The hybridization results in a shift in the resonance wavelength (Figure 18c). The multiplexing capability was demonstrated in an experiment with four arrays individually functionalized on the same chip with unique ssDNA complementary to four different miRNAs (iR-133b, miR-21, miR-24-1, and let-7c). The miRNA specific responses were observed at the microring resonators when the appropriate complementary miRNA solution flowed over the sensor array and the biorecognition/ hybridization event was clearly observed. The LOD of the microring resonator system was 150 fM without any amplification step and with a response time as low as 10 minutes. In order to improve the sensitivity of the biosensor, an amplification step using anti-DNA–RNA antibodies was incorporated to achieve the LOD microRNAs at 10 pM [78]. In the amplification step, the anti-DNA–RNA antibody attached to the heteroduplexes selectively (Figure 19), thereby resulting in an overall mass increase in the duplexes via the antibody (~150 kDa) and a larger optical signal than those achieved with the original miRNA (~7 kDa) hybridization.

## 5. Conclusions

The label-free and amplification-free ultrasensitive determination of miRNA is extremely challenging. Most of the conventional clinical methods used to analyze microRNA require sample transportation, laborious preparation steps, and skilled end-users. Although there are very limited reports on the development of label-free miRNA based optical biosensors, the scientific literature shows that there is a great deal of interest in miRNA assay development and this can be expected to continue to grow at an exponential rate. Label-free microRNA optical sensors provide rapid and cost-effective approaches to achieve high sensitivity and LOD. A few groups have already demonstrated the promising approaches of the optical miRNA biosensors by integrating nanomaterials due to their unique optical properties and ability to enhance the optical signal. However, the addition of an amplification step further complicates the sensor development process where multiple preparation and detection steps are required.

It has been demonstrated that the label-free enzyme and DNA-coated AuNPs amplification strategy employed using SPRi can be used to detect and quantify miRNAs down to fM levels with very good sensitivity. Even lower limits of detection have been achieved by incorporating signal amplification strategy down to the aM level using a more complicated assay format. The tapered microfiber aligned silica capillary is one of the most cost effective, easy to fabricate optical biosensors that offers a great degree of portability. Microring resonators have been successfully demonstrated to achieve extremely low LOD for single molecule, however it is ultrasensitive to the point where the optical signal is impacted by environmental conditions such as temperature, humility, electric or magnetic field, etc. SPR based biosensors can offer sensitive, fast, and on-site analysis, and therefore, they can be integrated with smart phone platforms in the development of POC systems for miRNA detection and can serve as an alternative to conventional techniques.

The ability to measure miRNA at ultralow concentration levels with high selectivity in a variety of biological samples is of great interest in clinical diagnostics and remains to be fully explored for human specimens such as tissues, blood, or urine directly. The ideal optical biosensor for miRNA will require a minimum or no sample preparation or pretreatment and will provide a great opportunity for the early diagnosis of many diseases. Future research directions in this field may involve the implementation of multiplexable arrays for the high throughput screening and complete profiling of miRNA in biological relevant samples. Optical based biosensors have the potential to fully unravel miRNA biological mechanisms and functions and to enable the development robust POC diagnostic systems.

## Figures and Tables

**Table 1:** Comparison of label-free microRNA optical sensors.

miRNA Type	Sample Source	Method	LOD	Sample Volume	Reference
miR-16, miR-23b, and miR-122b	mouse liver tissue	SPRi	10 fM	500 µL	Fang 2006 [77]
miR-133b, miR-21, miR-24-1, and let-7c	U87 MG cells	microrings	150 fM	75 µL.	Qavi 2010 [73]
miR-16, miR-21, miR-24-1, and miR-26a	total mouse brain RNA	microrings	10 pM	35 μL	Qavi 2011 [78]
miR-21, and let-7a	clinical urine samples	MZI	1 nM	70 μL (PBS) 200 mL (urine)	Liu 2015 [31]
miR-let7a	synthetic RNA	MZI	202 pM	--	Liang 2017 [79]
miR-122	mouse liver tissue	SPR	2 pM	440 μL	Sipova 2010 [71]
miR-21 and miR-10b	human plasma	LSPR	23–35 fM	50 µL	Joshi 2014 [62]
miR-10b	human plasma	LSPR	aM	2.5 mL	Joshi 2015 [63]
miR-15a	Human plasma	SPRi	0.56 fM		Hu 2017 [75]
miR-93	synthetic RNA	SPR	10 pM	25 μl	Schmieder 2016 [25]
miR-21and miR-155	synthetic RNA	LSPR	10 aM	5 μL	Xue 2019 [44]
